# Identifification and validation of ferroptosis signatures and immune infifiltration characteristics associated with intervertebral disc degeneration

**DOI:** 10.3389/fgene.2023.1133615

**Published:** 2023-02-22

**Authors:** Feng Zhang, Di Cui, Kangkang Wang, Huimin Cheng, Yunlei Zhai, Wei Jiao, Zhaodong Wang, Xilong Cui, Haiyang Yu

**Affiliations:** ^1^ Department of Orthopedics, Affiliated Fuyang People’s Hospital of Anhui Medical University, Fuyang, Anhui, China; ^2^ Clinical Research Center for Spinal Deformity of Anhui Province, Fuyang, Anhui, China; ^3^ Medical School of Fuyang Normal University, Fuyang, Anhui, China; ^4^ Anhui Province Key Laboratory of Tissue Transplantation, Bengbu Medical College, Bengbu, Anhui, China; ^5^ Department of Orthopedics, the First Affiliated Hospital of Bengbu Medical College, Bengbu, Anhui, China

**Keywords:** intervertebral disc degeneration, ferroptosis, immune infifiltration, NOX4, ENPP2

## Abstract

Ferroptosis and immune infiltration play an important role in the pathogenesis of intervertebral disc degeneration (IDD). However, there is still a lack of comprehensive analysis on the interaction between ferroptosis-related genes (FRGs) and immune microenvironment in IDD patients. Therefore, this study aims to explore the correlation between FRGs characteristics and immune infiltration in the progression of IDD. The expression profiles (GSE56081 and GSE70362) and FRGs were downloaded from the comprehensive gene expression omnibus (GEO) and FerrDb database, respectively, and the differences were analyzed using R. The intersection of IDD related differential genes (DEGs) and FRGs was taken as differentially expressed FRGs (DE-FRGs) and GO and KEGG enrichment analysis was conducted. Then, we used least absolute shrinkage and selection operator (LASSO) regression algorithm and support vector machine (SVM) algorithm to screen feature genes and draw ROC curve judge the diagnostic value of key DE-FRGs. Then CIBERSORT algorithm is used to evaluate the infiltration of immune cells and analyze the correlation between key DE-FRGs and immune infiltration. Based on the analysis results, we conducted single gene GSEA analysis on key DE-FRGs. RT-PCR and immunohistochemistry further verified the clinical value of the results of biochemical analysis and screening. Seven key DE-FRGs were screened, including the upregulated genes NOX4 and PIR, and the downregulated genes TIMM9, ATF3, ENPP2, FADS2 and TFAP2A. Single gene GSEA analysis further elucidates the role of DE-FRGs in IDD associated with ferroptosis. Correlation analysis showed that seven key DE-FRGs were closely related to immune infiltration in the development of IDD. Finally, RT-PCR and immunohistochemical staining showed that NOX4, ENPP2, FADS2 and TFAP2A were statistically significant differences. In this study, we explored the connection between ferroptosis related characteristics and immune infiltration in IDD, and confirmed that NOX4, ENPP2, FADS2, and TFAP2A may become biomarkers and potential therapeutic targets for IDD.

## 1 Introduction

Lower back pain (LBP) is one of the most common complaints and the main cause of disability worldwide (Hartvigsen et al., 2018; Cieza et al., 2021). Intervertebral disc degeneration (IDD) is the most common cause of LBP (Guo et al., 2020). It has a significant impact on life quality and places a heavy load on the global healthcare system (Andersson, 1999). There has been little improvement in the early diagnosis of IDD, which currently still mostly relies on clinical symptoms and imaging findings. Although conservative therapy and surgical therapy are now thought to be effective ways to reduce the suffering of IDD patients, these therapies can only lessen the severity of symptoms which cann’t treat the underlying cause of the disease (Ma et al., 2022). As a result, early biomarker screening is important for IDD diagnosis and treatment. This cann’t only better protect the intervertebral discs’ biological function but also lessen the likelihood that individuals will get LBP.

Intervertebral disc (IVD) is composed of nucleus pulposus (NP), annulus fibrosus (AF) and cartilage endplate (CEP) (Maitre et al., 2015). Previous studies have reported that the main factors of IDD are genetic factors, aging, malnutrition and overload history (Sharifi et al., 2015). More and more studies showed that the immune system have played an important role in the progress of IDD (Capossela et al., 2014; Ye et al., 2022). NP is isolated from the host’s immune system by surrounding AF and CEP and becomes an immune privileged organ. Once this complete structure is destroyed, NP will be exposed to the immune system, further damaging the internal environment of the disc and causing a series of immune reactions (Sun Z et al., 2020). The activation of infiltrating immune cells, including macrophages (Silva et al., 2019) and CD8^+^T cells (Ye et al., 2022) in the intervertebral disc microenvironment help to accelerate the process of IDD. So far, only a few studies on immune infiltration in IDD development have been reported (Wang et al., 2021; Li K et al., 2022). Ferroptosis can regulate cell death by accumulation of iron-dependent lipid peroxides and reactive oxygen species (ROS) (Dixon et al., 2012). Current studies have found that ferroptosis is involved in the IDD process (Lu et al., 2021; Yang et al., 2021; Zhang et al., 2021), which may be an important factor of IDD. At present, the mechanism of ferroptosis in IDD and its relationship with immune infiltration remain unclear.

In this study, the key differentially expressed ferroptosis-related genes (DE-FRGs) were selected by bioinformatics analysis, and they were comprehensively analyzed by functional and enrichment analysis and immunoinfiltration analysis. Following that, machine learning models, expression validation across several data sets, and ROC curve analysis were used to assess the dependability of these crucial DE-FRGs. Finally, RT-PCR and immunohistochemistry were used to further corroborate the results. By integrating ferroptosis and immunological infiltration, we expect to offer a fresh viewpoint on the diagnosis and therapy of IDD.

## 2 Materials and methods

### 2.1 Data collection

**FIGURE 1:**
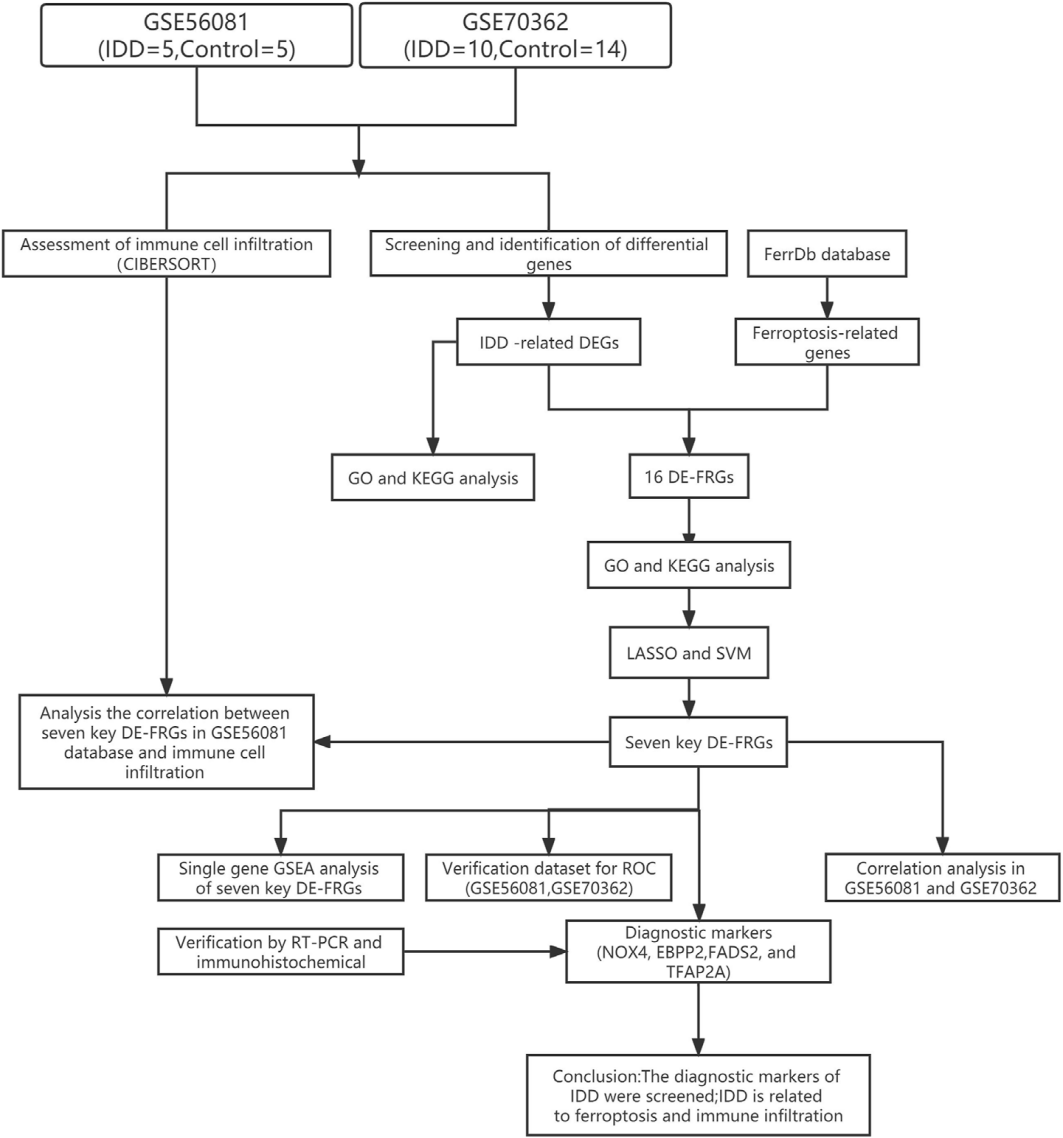
The flowchart of the analysis process.

In the Gene Expression Omnibus (GEO), with intervertebral disc degeneration and nucleus pulposus cells as the retrieval conditions, the species was set as human, and finally two datasets (GSE56081 and GSE70362) were selected for analysis. GSE56081 contains five nucleus pulposus samples from IDD patients and five normal nucleus pulposus samples. GSE70362 included samples from 10 IDD patients and 14 normal nucleus pulposus samples. The two datasets were normalized using the “preprocessCore” package. In addition, cell classification inference of immune cell composition was performed in both the initial and validation datasets, allowing the immunomodulatory effects of key IDD biomarkers to be compared across different IDD samples. The ferroptosis-related genes (FRGs) were derived from FerrDb database. The flowchart of the analysis process was summarized in Figure 1.

### 2.2 Screening and identification of differential genes

The “preprocessCore” packages in R software were used to avoid batch effects, and the normalization of both datasets was validated by boxplots with the ggpolt2 package. Differential genes (DEGs) were determined using the limma software package analysis, with |log2 (fold change)|>1 and *p* < 0.05 as DEGs with statistically significant differences. Volcano plots and heatmaps were generated using “ggplot2” and the “pheatmap” packages, showing statistically significant DEGs. Intersection of FRGs with DEGs using the Venn diagram package and showing the expression levels of DE-FRGs by drawing heatmap.

### 2.3 Gene ontology (GO) and kyoto encyclopedia of genes and genomes (KEGG) pathway analysis of DEGs

To further reveal the important functions and biological pathways of co-expressed differentially genes, we performed GO function and KEGG pathway enrichment analysis on them. The “clusterProfiler”, “org.Hs.e.g.db”, and “ggplot2” packages in the R software were used to analyze and visualize the GO and KEGG pathway enrichment of differential genes. Setting the minimum gene set to five and the maximum gene set to 5000, *p* values of <0.05 and FDR of <0.25 were considered statistically significant.

### 2.4 Screening and identification of key genes

In order to further identify key genes from differentially expressed ferroptosis-related genes, the Lasso regression algorithm and the SVM-RFE support vector machine recursive feature elimination algorithm were used to screen eigengenes. The gene results were intersected, and the R software “venneuler” package was used to draw a Venn diagram to visualize the results.

### 2.5 Validation of diagnostic markers

For the eigengenes obtained by the above intersection, the value of the obtained genes as diagnostic markers was verified in two independent data sets, GSE56081 and GSE70362, and the diagnostic value was evaluated by drawing the receiver operating characteristic (ROC) curve. With *p* < 0.05 is the threshold to determine.

### 2.6 Assessment of immune cell infiltration

In order to evaluate the infiltration of immune cells in IDD and the relationship between the gene expression of the screened IDD diagnostic biomarkers and the infiltration of immune cells in the intervertebral disc tissue, the CIBERSORT algorithm (https://cibersort.stanford.edu/) was used to analyze the GSE56081 and GSE70362, and the appropriate samples were screened according to *p* < 0.05 and the percentage of each type of immune cell in the sample was calculated. Wilcox test was used to compare the differences between the two groups, and the correlation between the infiltration rates of various types of immune cells was determined through the corrplot package.

### 2.7 Human NP tissue sample collection

This study used NP tissue, which had the informed consent of the patient’s family and was approved by the Medical Ethics Committee of Fuyang People’s Hospital (Fuyang, China). Ethical approval number is Review of Medical Ethics [2022]2. During the period from January 2022 to August 2022, the degenerative samples were taken from the degenerative NP cell samples of patients with degenerative intervertebral disc disease who underwent discectomy in Fuyang People’s Hospital, and the control samples were taken from the patients who had undergone surgery for idiopathic scoliosis. After cell collection, NP cells were resuspended in DMEM/F12 medium containing 10% FBS for culture. Observe the cell growth and adherence every day, and pass it to the third generation for relevant experiments. The clinical characteristics of the patients are shown in Table 1.

**TABLE 1:** Clinical data of patients.

Pfirrmann	Age (year)	Gender (male/female)	Diagnosis	Lumbar level
Ⅰ	14	Male	Idiopathic scoliosis	T11/12
	16	Female	Idiopathic scoliosis	T12/L1
Ⅱ	22	Male	Lumbar disc herniation	L4/5
	28	Male	Lumbar disc herniation	L3/4
Ⅲ	46	Female	Lumbar disc herniation	L4/5
	28	Male	Lumbar disc herniation	L4/5
Ⅳ	51	Female	Lumbar disc herniation	L4/5
	53	Female	Lumbar disc herniation	L4/5
Ⅴ	65	Female	Lumbar spondylolisthesis	L4/5
	79	Female	Lumbarn disc herniation	L4/5

### 2.8 Real time polymerase chain reaction (RT-PCR)

The primer sequence of each gene was shown in Table 2. A total of 10 NP tissues were used, of which 5 were degenerative and five were normal. Nucleus pulposus cells are digested by trypsin and treated with the TriZol reagent (Invitrogen) to extract total RNA after ultrasonic fragmentation. The PrimeScript RT-PCR kit (Takara) was used to synthesize cDNA. The 7500 real-time fluorescent PCR system (Thermo Fisher) was used for RT-PCR, and GAPDH was used as the endogenous control.

**TABLE 2:** Sequences of the RT-PCR primers used for each gene.

Gene	Forward primer sequence	Reverse primer sequence
FADS2	TGA​CCG​CAA​GGT​TTA​CAA​CAT	AGG​CAT​CCG​TTG​CAT​CTT​CTC
PIR	GAG​CAG​TCG​GAA​GGG​GTT​G	TTA​ACT​CGG​GTC​TGC​CAA​TGC
NOX4	CAG​ATG​TTG​GGG​CTA​GGA​TTG	GAG​TGT​TCG​GCA​CAT​GGG​TA
ENPP2	ACT​TTT​GCC​GTT​GGA​GTC​AAT	GGA​GTC​TGA​TAG​CAC​TGT​AGG​A
TFAP2A	AGG​TCA​ATC​TCC​CTA​CAC​GAG	GGA​GTA​AGG​ATC​TTG​CGA​CTG​G
TIMM9	AGA​GAG​CAG​TGC​AGG​ATG​TG	GGC​AGT​TGA​GGC​ATT​GAA​CC
ATF3	CCT​CTG​CGC​TGG​AAT​CAG​TC	TTC​TTT​CTC​GTC​GCC​TCT​TTT​T
GAPDH	GGA​GCG​AGA​TCC​CTC​CAA​AAT	GGC​TGT​TGT​CAT​ACT​TCT​CAT​GG

### 2.9 Immunohistological analyses in human NP tissues

The expression of DE-FRGs was evaluated by immunohistochemistry of sections of nucleus pulposus with different levels of degeneration. NP tissue was fixed with 4% paraformaldehyde, then paraffin embedded and sectioned. Histological analysis was performed. Sections were desaffinified, rehydrated, and stained with Eosin (HE), Masson, and Alcian blue, respectively. Immunohistochemistry was performed according to the aforementioned methods (Yi et al., 2020). Sections were incubated with a primary antibody (dilution 1:200) resistant to ENPP2 (14243-1-AP, Wuhan proteintech), NOX4 (14347-1-AP, Wuhan proteintech), FADS2 (680261-LG, Wuhan proteintech), and TFAP2A (67076-1-lg, Wuhan proteintech). Next, the slices were incubated with the secondary antibody (pv6000, Beijing Zhongshan). Buffer was used instead of primary antibody as negative control. Finally, three fields of each slide were randomly selected and observed with a microscope (Olympus, Tokyo, Japan).

### 2.10 Statistical analysis

Analyze the statistical data obtained from GEO database through R-3.6.1. Use GraphPad Prism 8 and SPAS software to process other data. *p* < 0.05 was considered statistically significant.

## 3 Results

### 3.1 Identifification of IDD-related genes

**FIGURE 2:**
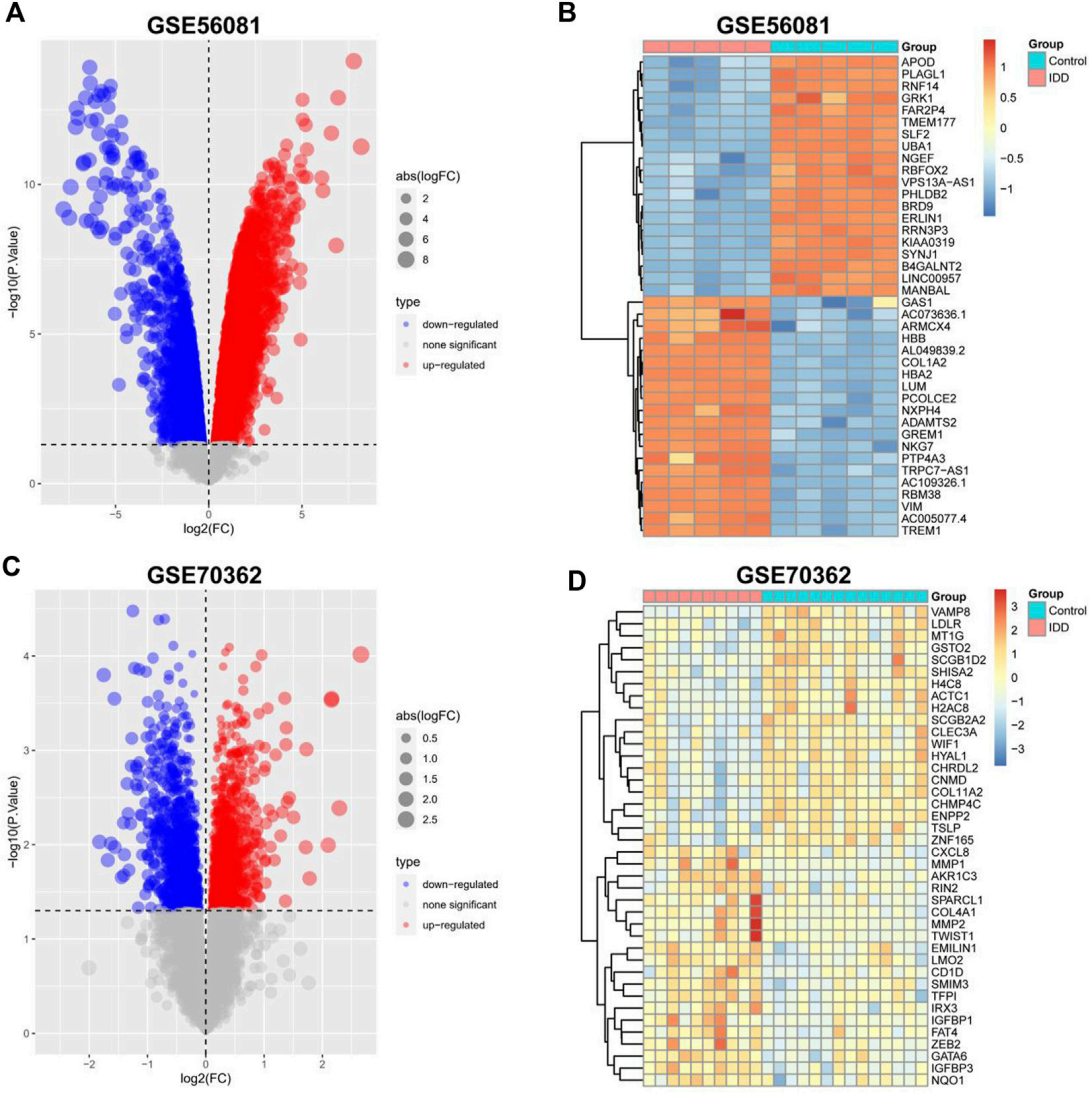
Identification of differentially expressed IDD-related genes. **(A,C)** Volcano plot of DEGs between intervertebral disc degeneration and normal control group. **(B,D)** Heatmap of DEGs between intervertebral disc degeneration and normal control group.

**FIGURE 3:**
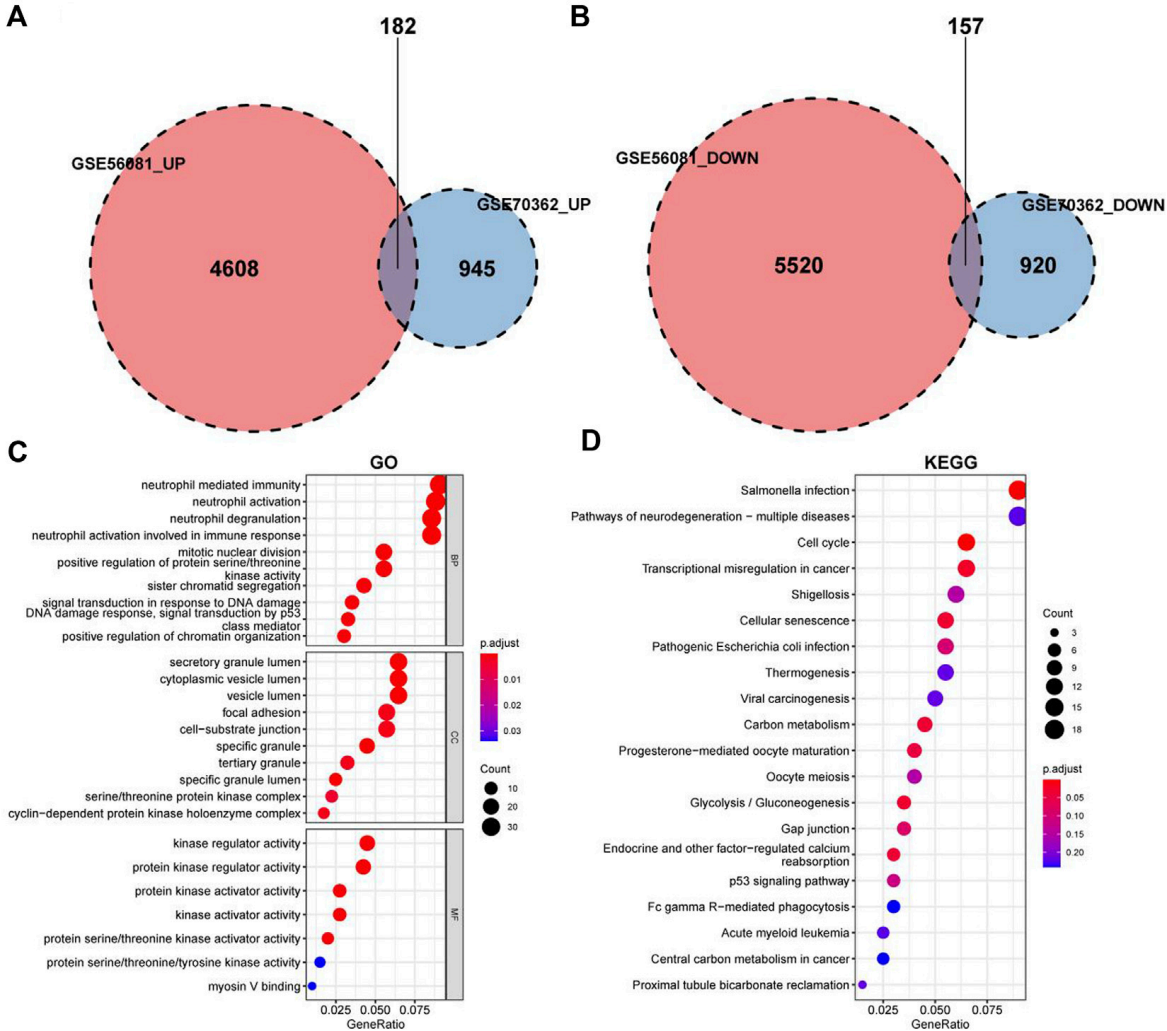
GO and KEGG enrichment analysis for the IDD-related DEGs. **(A)** Differential analysis upregulated gene intersection in GSE56081 and GSE70362. **(B)** Differential gene downregulation gene intersection in GSE56081 and GSE70362. **(C)** GO terms enriched by the differentially expressed IDD-related DEGs (MF:molecular function, BP:biological process and CC:cellular component). **(D)** KEGG pathways enriched by the differentially expressed IDD-related DEGs.

The data sets GSE56081 and GSE70362 were homogenized using the “preprocesscore” package (Supplementary Figures S1A, B). Next, we used the “limma” package to analyze the differences between the two datasets. A total of 339 differential genes were screened, including 182 upregulated genes and 157 downregulated genes. Figures 2A, C show the volcano map of differential genes analysis, while Figures 2B, D show the analysis heat map of differential genes. To explore the biological function of the differential genes related to IDD, we performed GO and KEGG enrichment analysis. The results of GO analysis showed that these differential genes were mainly related to neutrophil mediated immunity, neutrophil activation involved in immune response, mitotic nuclear division and so on (Figure 3C). In addition, the KEGG enrichment pathway analysis shows multiple important signaling pathways such as pathways neurodegeneration-multiple.

### 3.2 Identification of DE-FRGs

**FIGURE 4:**
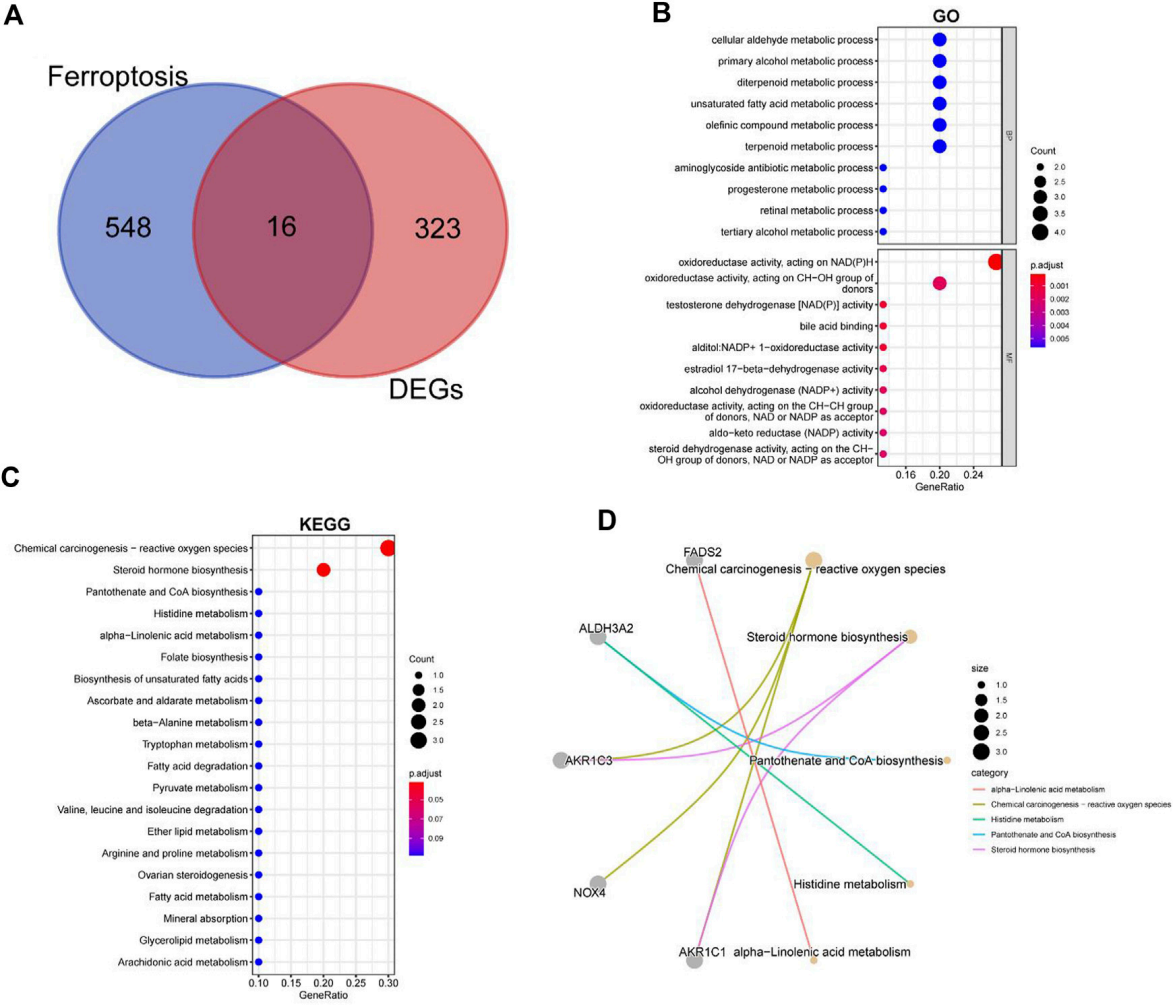
GO and KEGG enrichment analysis for the ferroptosis-related DEGs. **(A)** Intersection of ferroptosis-related genes and IDD-related genes. **(B)** GO terms enriched by the differentially expressed ferroptosis-related DEGs. **(C)** KEGG pathways enriched by the differentially expressed ferroptosis-related DEGs. **(D)** The relationship between KEGG results of top5 and its related DEGs.

In order to explore the relationship between ferroptosis and intervertebral disc degeneration, we crossed the previously obtained differential genes with FRGs and obtained a total of 16 DE-FRGs (Figure 4A). Supplementary Figures S2 shows the volcanic map and heatmap of ferroptosis related differential genes in GSE56081 and GSE70362 separately. The enrichment results of GO and KEGG showed that the DE-FRGs were closely related to cellular aldehyde metabolic process, oxidoreductase activity and chemical carcinogenesis-reactive oxygen species (Figures 4B, C). In addition, we use node graph to show the relationship between KEGG results of top5 and related differential genes (Figure 4D).

### 3.3 Screening key differential genes of IDD by machine learning

**FIGURE 5:**
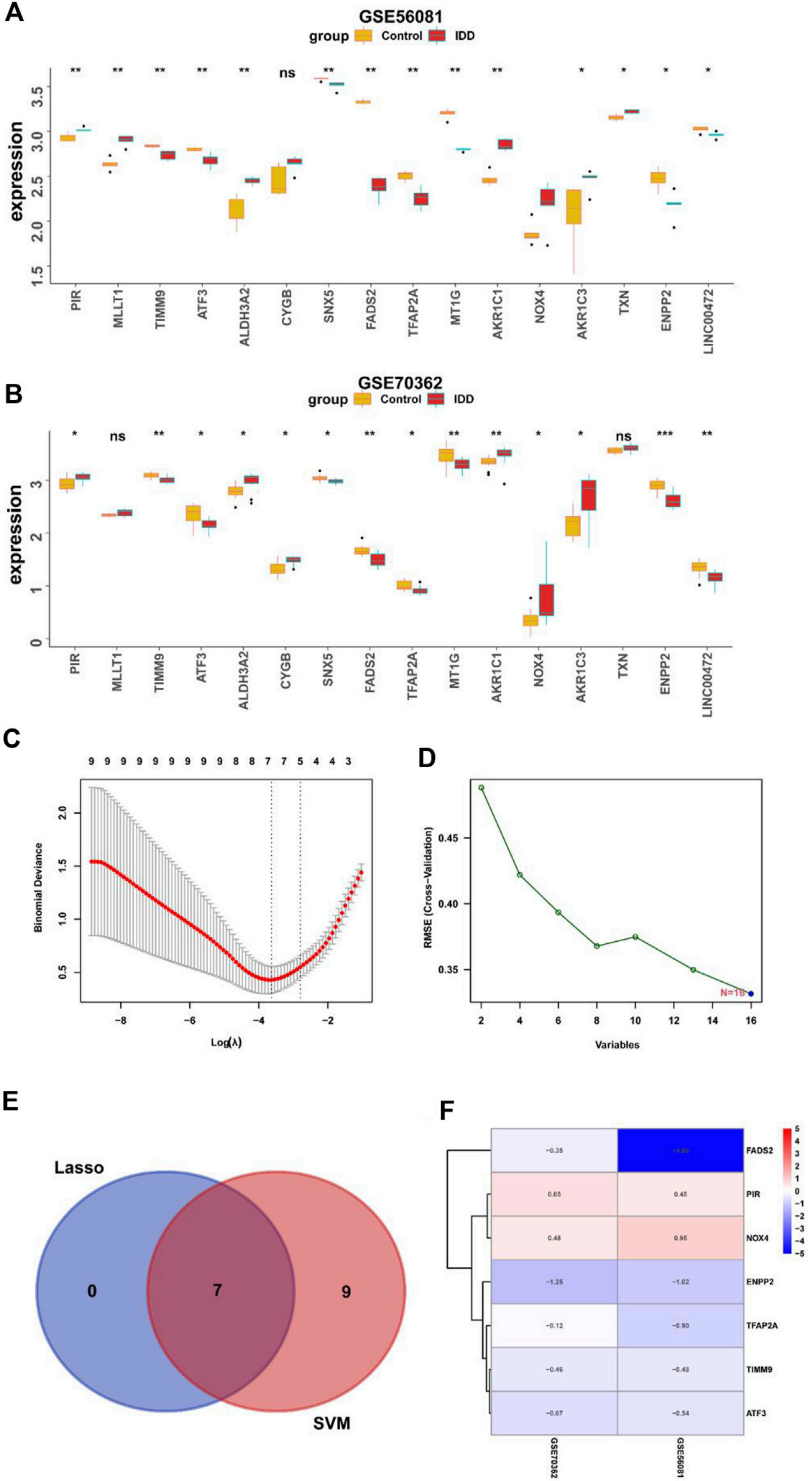
Screening key DEGs by machine learning. **(A)** Expression of ferroptosis-related DEGs in GSE56081 (*, *p* < 0.05; **,*p* < 0.01). **(B)** Expression of ferroptosis-related DEGs in GSE70362 (*, *p* < 0.05; **,*p* < 0.01). **(C)** Lasso algorithm to screen characteristic genes. **(D)** Support vector machine-recursive feature elimination algorithm to screen characteristic genes. **(E)** Venn diagram showed the overlapped genes obtained by the LASSO algorithm and SVM-RFE algorithm. **(F)** LogFC values of key DEGs in the difference analysis results of two datasets.

Next, we analyzed the differential expression of 16 DE-FRGs in two data sets. As shown in Figures 5A, B genes were upregulated, and nine genes were downregulated in two databases. In order to further identify the key genes, we further screen the characteristic genes through machine learning. Seven genes were screened by LASSO analysis and 16 genes were screened by SVM algorithm. Seven feature genes were obtained through LASSO algorithm and SVM algorithm, and 16 feature genes are obtained through SVM-RFE algorithm (Figures 5C, D). The genes obtained by the two methods were intersected to obtain seven characteristic marker genes, and the Wayne map was drawn (Figure 5E). Finally, heat map is used to display the logFC values of seven genes in the difference analysis results of two data sets, including two highly expressed genes and five low expressed genes (Figure 5F).

### 3.4 Correlation analysis and diagnostic value evaluation of key characteristic genes

**FIGURE 6:**
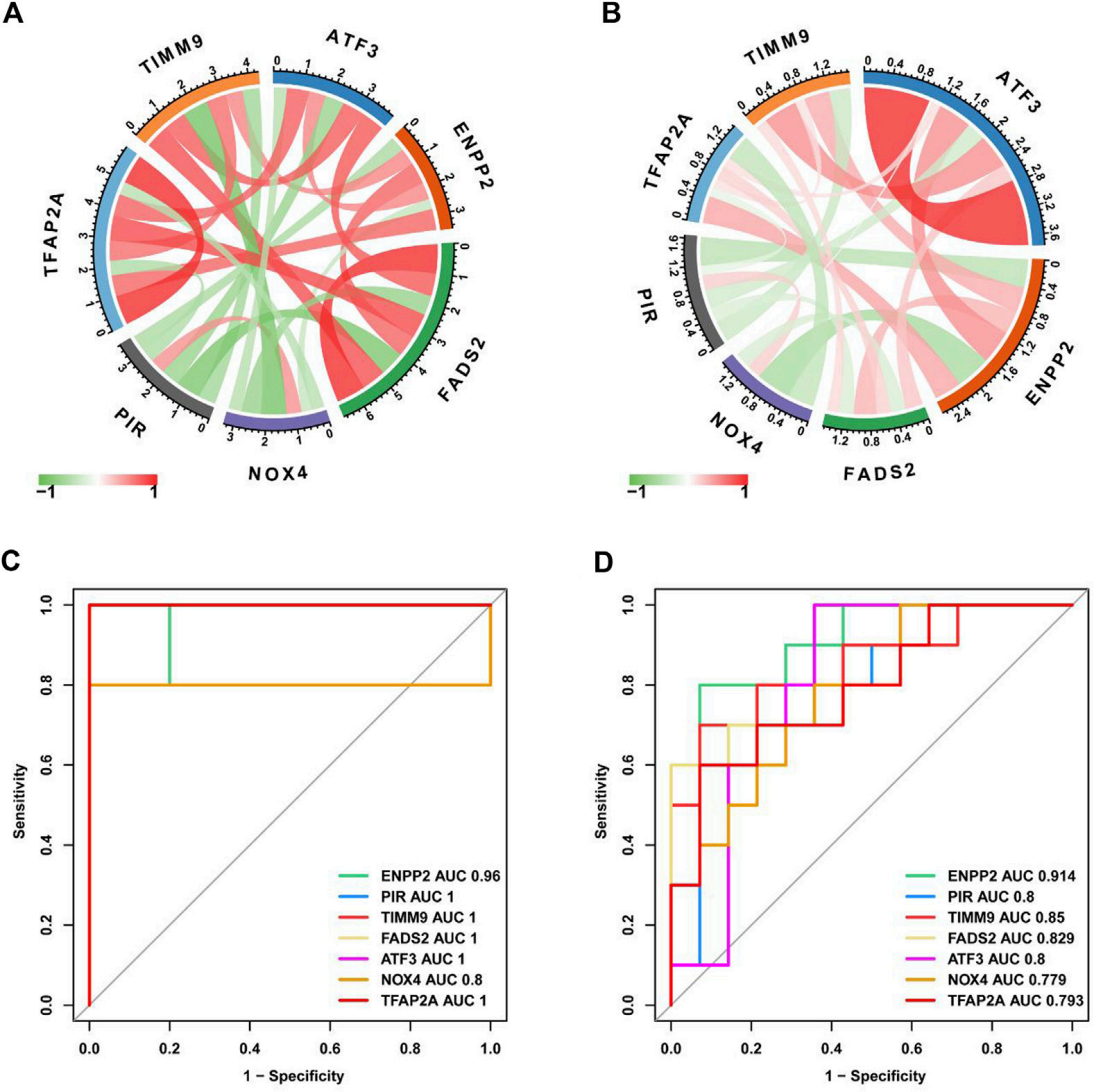
Evaluation of diagnostic value of key characteristic genes. **(A)** Correlation analysis of key DEGs in GSE56081 dataset. **(B)** Correlation analysis of key DEGs in GSE70362 dataset. **(C)** ROC curves for key DEGs in GSE56081 dataset. **(D)** ROC curves for key DEGs in GSE70362 dataset.

Next, we use the “circle” package to analyze the correlation of seven core genes in the two data sets. As shown in Figures 6A, B, NOX4 and PIR were negatively correlated with other genes, while TFAP2A, ATF3, ENPP2, FADS2 and TIMM9 were positively correlated with other genes. We drawed ROC curves and evaluate the diagnostic value of key characteristic genes by calculating AUC values. ROC curve results showed that ENPP2 in two data sets AUC values of are 0.96 and 0.914 respectively, PIR in two data AUC values of the set are 1.0 and 0.8 respectively, TIMM9 in two data AUC values of the set were 1.0 and 0.85 respectively. The results show that they have high diagnostic value in IDD (Figures 6C, D).

### 3.5 Immunocyte infiltration analysis

**FIGURE 7:**
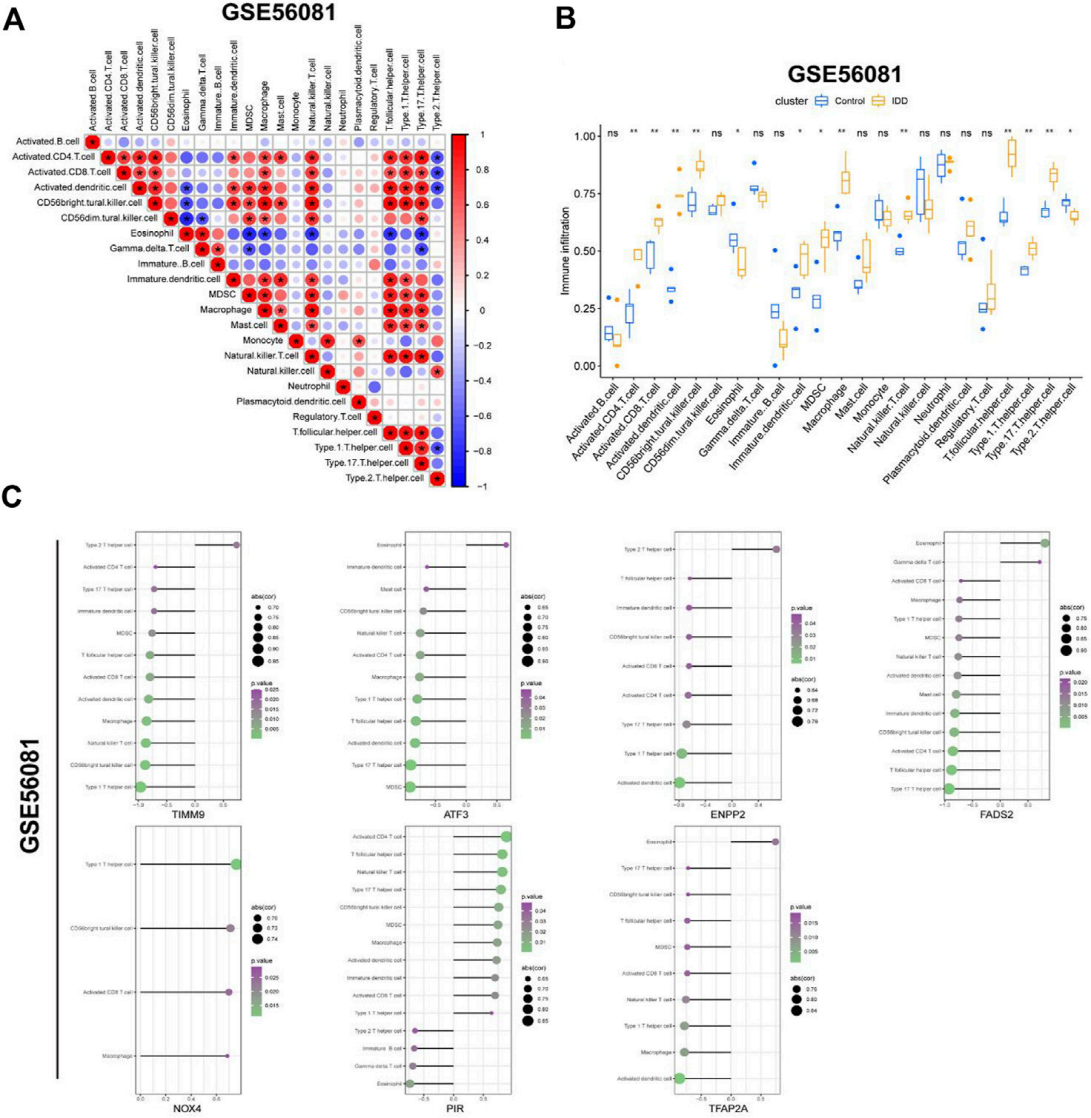
Evaluation of immune cell infiltration. **(A)** Correlation between immune cells in GSE56081 dataset. **(B)** Histogram showed the differences of infiltrating immune cells between IDD samples and control samples (*, *p* < 0.05; **,*p* < 0.01). **(C)** Correlation between the expression of key DEGs and the infiltration of immune cells.

In order to explore the connection between IDD and immune infiltration, we used the ssGSEA function of “GSVA” package to evaluate the degree of immune cell infiltration in GSE56081 dataset. Figure 7A showed the correlation between immune cells in IDD, macrophages and natural killer cells were positively correlated with many other immune cells including activated CD4 T cells and CD8 T cells. Figure 7B showed the difference in the infiltration of immune cells in normal intervertebral disc NP cells and degenerative intervertebral disc NP cells. The activated CD4 T cells, CD8 T cells, dendritic cells and so on were increased in degenerated NP cells, while eosinophil and type 2T helper cell were decreased in degenerated NP cells. Next, we use Cibersort algorithm to analyze the relationship between the expression of seven key DE-FRGs in GSE56081 database and immune cell infiltration (Figure 7C). The upregulated expression of NOX4 and PIR in IDD was positively correlated with multiple immune cells, while the downregulated expression of TIMM9, ATF3, ENPP2, FADS2 and TFAP2A were negatively correlated with multiple immune cells. These results suggest that high immune infiltration may play an important role in intervertebral disc degeneration.

### 3.6 Function enrichment analysis of key ferroptosis-related DEGs

**FIGURE 8:**
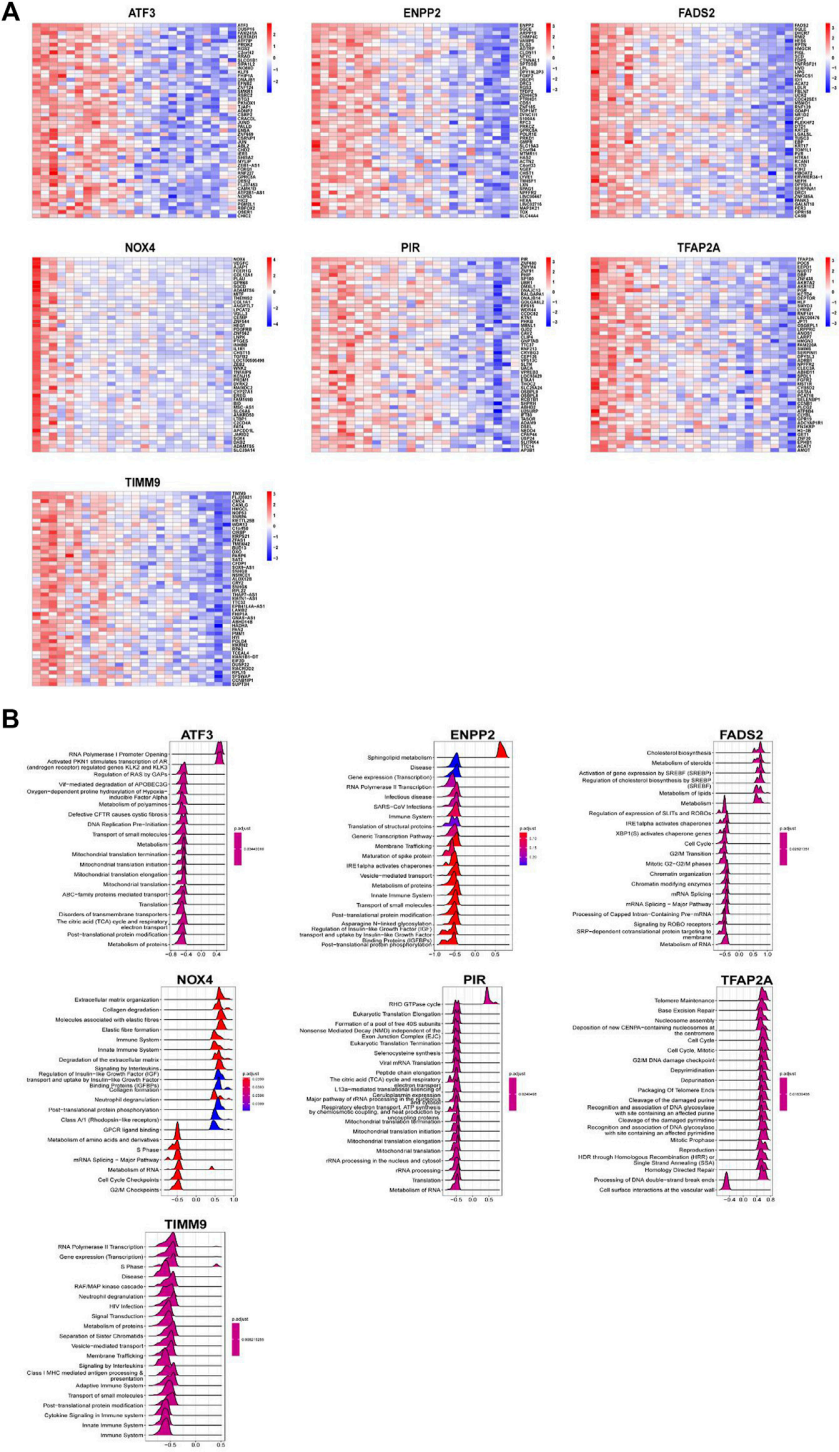
Function enrichment analysis of key DEGs. **(A)** Heatmap display of top50 gene positively related to key DEGs. Function enrichment analysis of key DEGs. **(B)** Top 20 of GSEA enrichment analysis results of key DEGs.

In order to further clarify the role of DE-FRGs in IDD, we conducted gene set enrichment analysis (GSEA) function enrichment analysis. Use the data in GSE70362 to analyze the correlation between seven key genes and all genes and use the heat map to display the positive correlation top50 gene respectively (Figure 8A). Based on the results of correlation analysis, we used the “clusterProfiler” package for single gene GSEA analysis of Reactome (Figure 8B). The enrichment results indicate that ATF3 and PIR may be related to the regulation of mitochondrial related genes. ENPP2, NOX4 and TIMM9 may be related to RNA transcription and immune system, while FADS2 and TFAP2A may participate in the IDD process by regulating the cycle of NP cells.

### 3.7 RT-PCR and immunohistological assessments validation of bioinformatics results

**FIGURE 9:**
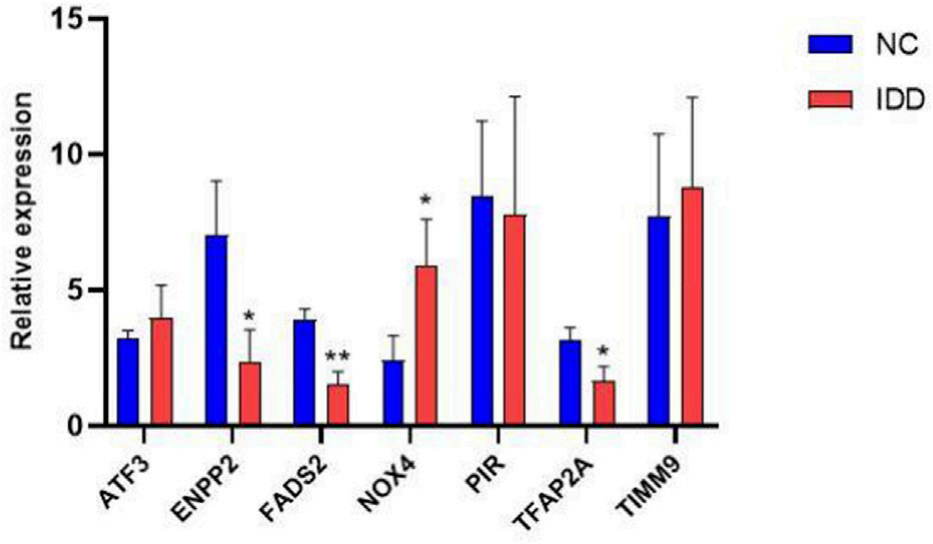
The mRNA relative expression levels of NOX4, ENPP2, FADS2 and TFAP2A in the IDD (*n* = 5) and normal groups (*n* = 5) were verified by RT-PCR operated, and the expression was calculated using the 2^−ΔΔCT^ method. The expression of NOX4, ENPP2, FADS2 and TFAP2A ware statistically significant (*, *p* < 0.05; **,*p* < 0.01), whares there ware no difference in ATF3, PIR and TIMM9 expression.

**FIGURE 10:**
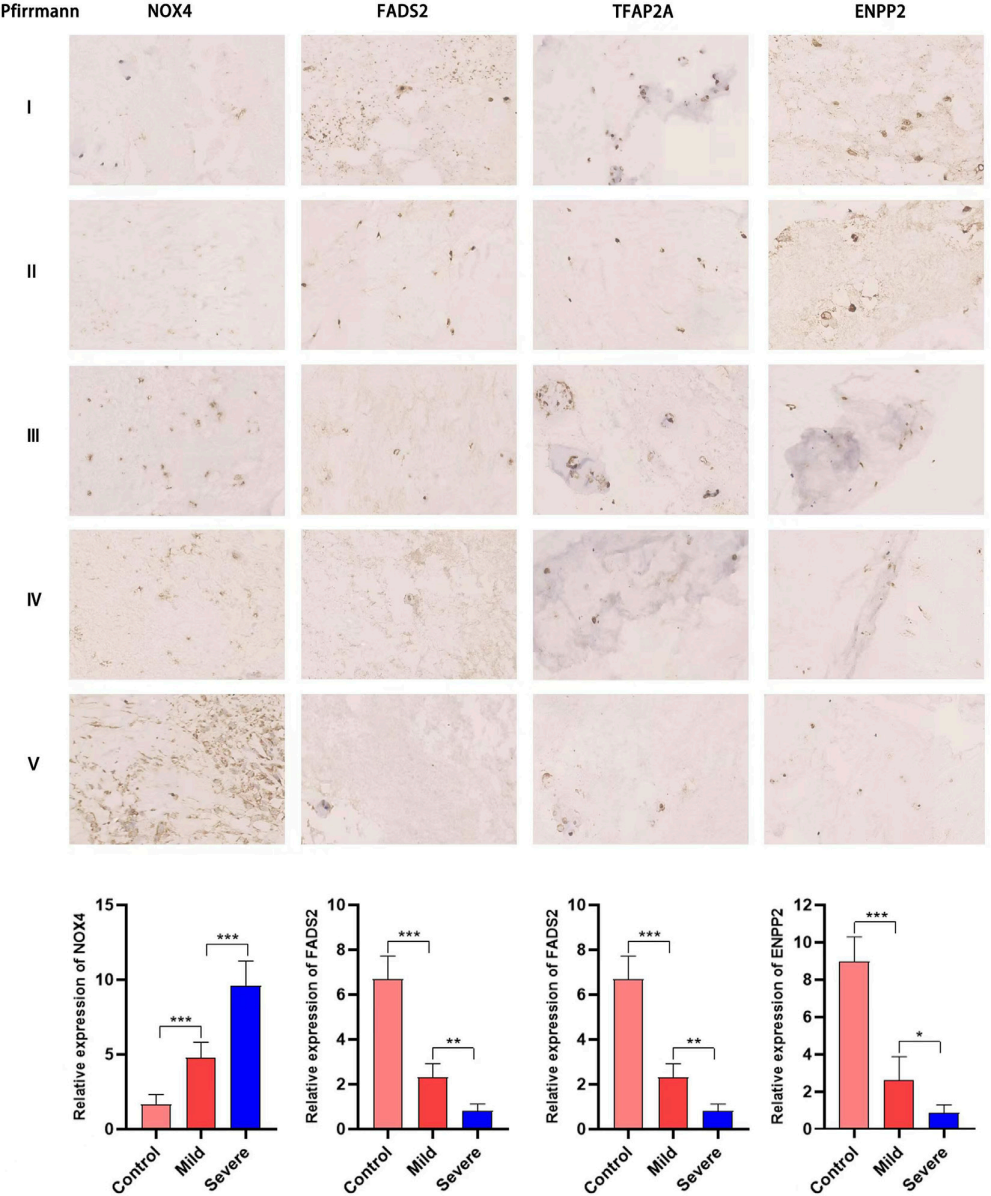
Immunohistochemical staining has proved that NOX4, FADS2, TFAP2A, ENPP2 expression in NP tissues of different degeneration grades. The expression of NOX4 increases with the increase of degeneration grade, while the expression of FADS2, TFAP2A, ENPP2 is gradually downregulated (*, *p* < 0.05; **,*p* < 0.01).

Next, we used RT-PCR and immunohistochemical staining to confirm whether key DE-FRGs can be applied non-selectively to IDD patients. RT-PCR experiment results showed that differences in the expression levels of ENPP2, NOX4, FADS2 and TFAP2A genes were statistically significant. ATF3, PIR and TIMM9 showed no statistically significant difference. The expression of NOX4 was upregulated, whereas the expression of ENPP2, FADS2 and TFAP2A was downregulated (Figure 9). We also confirmed the above results by immunohistochemistry (Figure 10). These results were consistent with the bioinformatics analysis. In addition, the MRI images of patients with different levels of degeneration were shown in Supplementary Figure S3A, and HE, Masson and Alxin blue staining of nucleus pulposus were shown in Supplementary Figure S3B.

## 4 Discussion

IDD is a protracted and gradual process that is influenced by oxidative stress, trauma, infection, inflammation, and other factors (Feng et al., 2016). The majority of patients with IDD are diagnosed in the late stages of degenerative changes because there are frequently no obvious clinical symptoms in the early stages, and the current clinical treatment strategy focuses more on managing their symptoms than on finding a solution for early diagnosis and prompt intervention. Ferroptosis is a type of regulatory cell death that depends on iron (Dixon et al., 2012). According to research, neurodegenerative disorders, cancer, stroke, and ischemia-reperfusion injury are all intimately related to ferroptosis (Reichert et al., 2020; Chen et al., 2021; Yan et al., 2021). Recent studies have shown that ferroptosis is involved in the process of IDD (Lu et al., 2021; Yang et al., 2021; Zhang et al., 2021), and immune infiltration plays an important role in the process of IDD (Wang et al., 2021; Li W et al., 2022). However, at present, there has been no comprehensive study on ferroptosis and immune infiltration in IDD. Therefore, we aimed to identify the ferroptosis signature gene in IDD and further investigate its correlation with immune infiltration.

Firstly, we analyze the differences between the two data sets, and a total of 339 differential genes were screened. The differential genes may have immune-related functions and may be strongly associated with IDD, according to GO enrichment analysis, which revealed that these differential genes were primarily connected to neutrophil activation, degranulation, and immunological response. The majority of white blood cells in the circulation are neutrophils, which are also the main effector cells of innate immunity (Lodge et al., 2020; Rosales, 2020). Neutrophils have the ability to produce and release chemokines, which can regulate acute injury and repair, cancer, autoimmune and chronic inflammatory processes (Tecchio and Cassatella, 2016; Liew and Kubes, 2019), and play an important role in the progression of IDD (Li W et al., 2022). The ssGSEA function of the R language’s “GSVA” package was used to assess the degree of immune cell infiltration in order to further assess the link between immune infiltration and IDD. The findings demonstrated that whereas eosinophils and Th2 cells reduced in the deteriorated nucleus pulposus compared to the normal group, the number of macrophages, CD8T cells, dendritic cells, and Th17 cells rose. The importance of macrophage infiltration in the etiology of IDD has been validated by recent research (Lan et al., 2022; Yan et al., 2022). According to Andrea et al., dendritic cells had a role in the immune response’s beginning in IDD, while macrophages further strengthened the immune response and controlled disc absorption (Geiss et al., 2016). Furthermore, th17 cells, a distinct subset of T helper cells, contribute to the pathophysiology of IDD by secreting interleukin-17 (Shamji et al., 2010). In addition, elevated levels of Th17 cells and interleukin-17 can exacerbate pain in IDD patients (Cheng et al., 2013). Our findings agree with reports in earlier literature. While this is going on, neutrophils, T cells, and macrophages can also emit cytokines including TNF-a, IL-1b, and IL-17 that accelerate IDD by encouraging the recruitment of immune cells into intervertebral disc tissues and the degradation of extracellular matrix (Risbud and Shapiro, 2014). Then, we used CIBERSORT algorithm to analyze the relationship between seven DE-FRGs and immune cell infiltration. We discovered that various immune cells are favorably correlated with NOX4 and PIR, which are highly expressed in IDD, while numerous immune cells are negatively correlated with TIMM9, ATF3, ENPP2, FADS2, and TFAP2A, which are low expressed. According to these data, increased immune infiltration might be a significant factor in disc degeneration.

Next, ferroptosis genes in IDD were screened for the first time using a combination of machine learning and immune infiltration, and a total of seven important genes, including NOX4, PIR, TFAP2A, ATF3, ENPP2, and TIMM9, were found. While TFAP2A, ATF3, ENPP2, FADS2, and TIMM9 are low expressed genes in IDD, NOX4 and PIR are highly expressed genes, suggesting that these seven genes may be involved in the ferroptosis process that leads to IDD. ROC curve was used to analyze the relationship between the sensitivity and specificity of these genes, and the results showed that the AUC values of these important genes were high, indicating that these genes had high diagnostic value. In order to further confirm the reliability of the bioinformatics analysis results, RT-PCR and immunohistochemistry were used for verification. The mRNA levels of ENPP2, NOX4, FADS2 and TFAP2A in IDD group and normal group were significantly different. The results of immunohistochemistry also confirmed the above results.

NADPH oxidase 4 (NOX4) is the main source of reactive oxygen species (ROS) and is expressed at elevated levels in animal models of IDD (Feng et al., 2017). Research shows that NOX4 promotes ferroptosis of astrocytes by lipid peroxidation induced by mitochondrial metabolic damage in Alzheimer’s disease (AD) (Park et al., 2021). Xiao et al. (Xiao et al., 2022) confirmed that NOX4, as one of the genes associated with ferroptosis, is an effective biomarker for the development of gastric cancer. In addition, NOX4 is also one of the marker genes of immune infiltration, and is highly correlated with the prediction of gastric cancer immunotherapy (Xin et al., 2022), the pathogenesis of keloid disorder (Yin et al., 2022), and the clinical outcome of colon cancer (Yang et al., 2020). Our results also confirm that NOX4 is positively correlated with the proportion of multiple immune cells, suggesting that NOX4 is very likely to accelerate the immune process of disc degeneration by promoting immune infiltration, but this needs to be verified by further molecular biology experiments. ENPP2, a member of the ecto-nucleotide pyrophosphatase/phosphodiesterase (ENPP) family, is known as autoprotein (ATX) (Borza et al., 2022). As a secretory enzyme, ENPP2 produces lysophosphatidic acid (LPA) signaling molecule, which significantly inhibits ROS production and ferroptosis in cardiomyocytes by regulating the expression of GPX4, ACSL4 and NRF2 (Bai et al., 2018; He et al., 2021). On the other hand, a recent study showed that ENPP2 could inhibit tumor infiltration of cytotoxic CD8^+^ T cells and thereby injure tumor regression (Matas-Rico et al., 2021). Therefore, ENPP2 may play a role in immune infiltration. Fatty acid desaturase 2 (FADS2) could fix in fatty acyl chain to adjust unsaturated fatty acids through the introduction of a double bond between carbon. Zhu et al. confirmed that FADS2 was correlated with immune infiltration of tumor cells through participation in peroxisome proliferator-activated receptors (PPARs) signaling through GSEA enrichment analysis (Zhu et al., 2021). Furthermore, interference with FADS2 expression could protect immortalized primary hepatocytes and lung cancer cells from erastin-induced ferroptosis. Transcription factor AP2 alpha (TFAP2A) is a transcription factor and also repress ferroptosis (Huang et al., 2020) and is associated with immune infiltration (Sun Y L et al., 2020).

In this study, machine learning was used to identify differential genes associated with ferroptosis, and CIBERSORT analysis was used to explore patterns of immune cell infiltration. Then the reliability of differential genes was evaluated by ROC curve and GSEA analysis of single genes. Finally, it was further verified by RT-PCR and immunohistochemistry. However, there are still limitations to this study. First of all, the database chosen for this study has a tiny sample size, which necessitates further research using larger samples. Second, this study is based on a secondary analysis of the information that has already been released. There should be some justifiable uncertainty regarding the validity of the data, even though they are largely consistent with results from earlier investigations.

## 5 Conclusion

Taken together, this study showed significant differences in the expression levels and immune infiltration of FRGs between IDD patients and healthy controls. Based on this comprehensive bioinformatic analysis, we identified the key genes, and immune infiltration characteristics of IDD. In addition, we confirmed the difference between key DE-FRGs in IDD patients using molecular biology experiments. Together, these findings may extend our understanding of ferroptosis and immune infiltration in patients with IDD.

## Data Availability

The original contributions presented in the study are included in the article/Supplementary Material, further inquiries can be directed to the corresponding authors.

## Abbreviations

IDD, Intervertebral disc degeneration; FRGs, Ferroptosis-related genes; GEO, Gene expression omnibus; DEGs, Difffferentially expressed genes; LASSO, Least absolute shrinkage and selection operator; SVM, Support vector machine; LBP, Low back pain; IVD, Intervertebral disc; AF, Annulus fifibrosus; NP, Nucleus pulposus; CEP, Cartilage endplate; ECM, Extracellular matrix; ROS, Reactive oxygen species; GO, Gene Ontology; KEGG, Kyoto Encyclopedia of Genes and Genomes; ROC, Receiver operating characteristic; GSEA, Gene set enrichment analysis.
