# Supplementary material for: Identifification and validation of ferroptosis signatures and immune infifiltration characteristics associated with intervertebral disc degeneration
Source: Front Genet. 2023 Feb 22;14:1133615. doi: 10.3389/fgene.2023.1133615 (PMC9992550; doi:10.3389/fgene.2023.1133615)
Supplement: Supplementary file 1 [file Image1.pdf]

Supplementary Material

Identifification and Validation of Ferroptosis Signatures and Immune Infifiltration Characteristics associated with Intervertebral Disc Degeneration

Feng Zhang[1,2], Di Cui[3], Kangkang Wang[1,2], Huimin Cheng[3], Yunlei Zhai[1,2], Wei Jiao[1,2],Zhaodong Wang[4,5]*,Xilong Cui[1,2]*,Haiyang Yu[1,2]*

* **Correspondence:** Haiyang Yu, fy.yhy@163.com; Xilong Cui, cuixilong.wang@163.com; Zhaodong Wang, wzd0703@163.com.

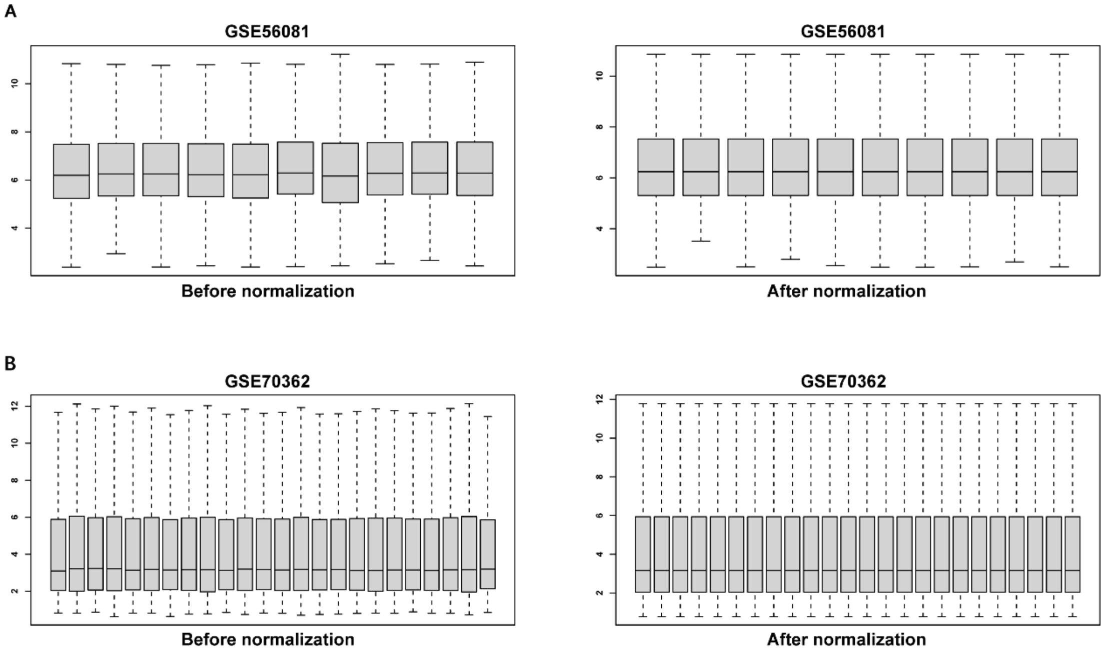

Supplement figure1: Homogeneous processing of gene expression data.A: Box plot of gene expression before and after homogenization in GSE56081.B: Box plot of gene expression before and after homogenization in GSE70362.

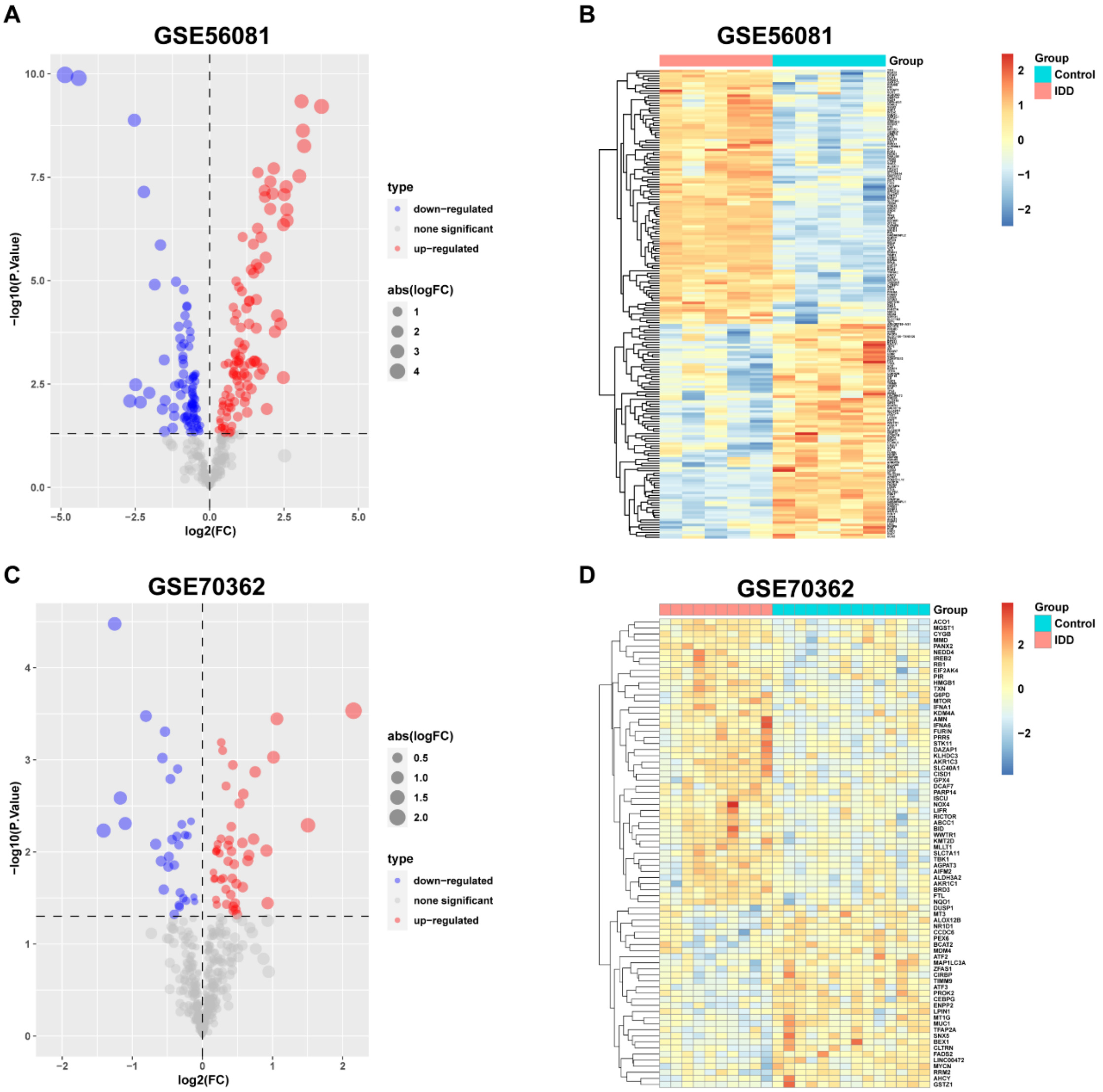

Supplement figure2: Identification of differentially expressed ferroptosis-DEGs. A,C: Volcano plot of ferroptosis-DEGs between intervertebral disc degeneration and normal control group. B,D: Heatmap of ferroptosis-DEGs between intervertebral disc degeneration and normal control group.

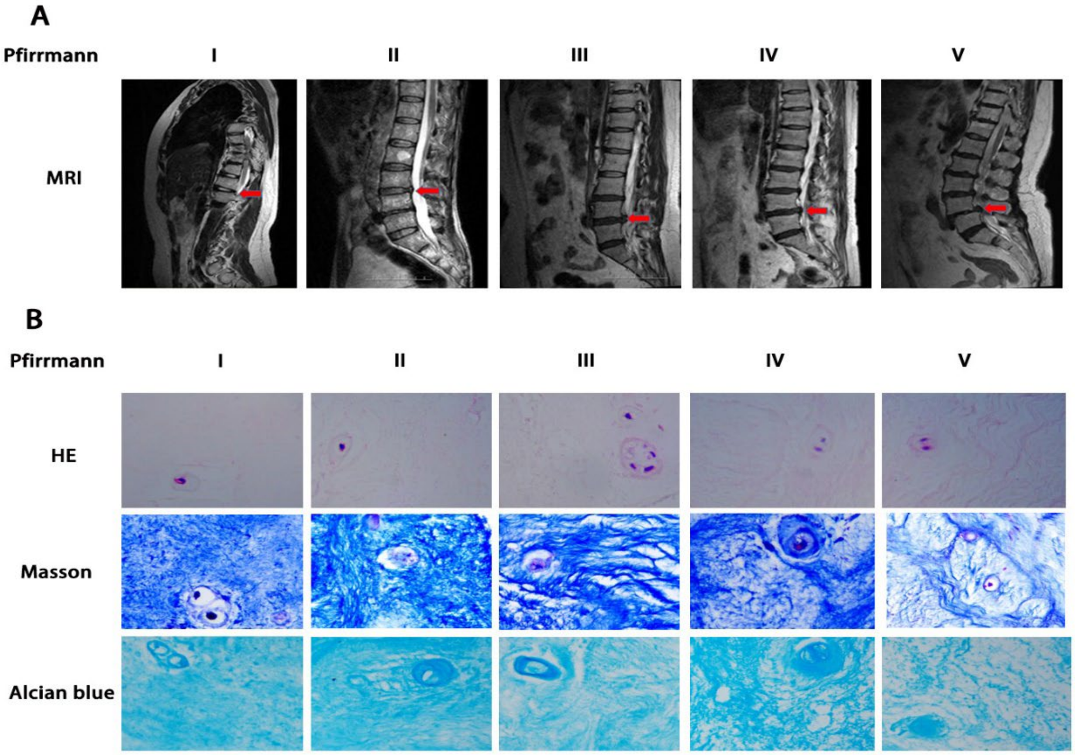

Supplement figure3: A: Spinal MRI images of patients with different levels of degeneration, the red arrow indicates the location of the lesion. B: HE, Masson and Alxin blue staining of nucleus pulposus.
